# Modifiable and emerging risk factors for type 2 diabetes in Africa: a systematic review and meta-analysis protocol

**DOI:** 10.1186/s13643-018-0801-y

**Published:** 2018-09-12

**Authors:** Ayuba Issaka, Yin Paradies, Christopher Stevenson

**Affiliations:** 1 School of Health and Social Development, Faculty of Health, Deakin University, 221 Burwood Highway, Burwood, Victoria 3125 Australia; 2 Alfred Deakin Institute, Faculty of Arts and Education, Deakin University, 221 Burwood Highway, Burwood, Victoria 3125 Australia

**Keywords:** Type 2 diabetes, Modifiable risk factors, Africa, Meta-analysis, Association

## Abstract

**Background:**

Type 2 diabetes mellitus (T2DM) remains a public health problem in low-income countries, including African countries. Risk factors of this disease in Africa are still unclear. This study will examine the modifiable and emerging risk factors associated with T2DM in Africa.

**Methodology:**

The study will include a systematic review and meta-analysis of published and unpublished empirical studies, reporting quantitative data only. We will conduct a search on scientific databases (e.g. Global Health), general online search engines (e.g. Google Scholar) and key websites for grey literature using a combination of key countries/geographic terms, risk factors (e.g. overweight/obesity) and T2DM (including a manual search of the included reference lists). We will use the Comprehensive Meta-Analysis Software (CMA) version 2.0 for data management and analysis. This protocol follows the Preferred Reporting Items for Systematic Review and Meta-Analysis Protocols (PRISMA-P).

**Discussion:**

The systematic review and meta-analysis will provide a robust and reliable evidence base for policy makers and future research. This may help with identifying and implementing more cost-effective diabetes prevention strategies and improved resource allocation.

**Systematic review registration:**

This protocol has been registered with the PROSPERO international prospective register of systematic reviews. The reference number is CRD42016043027.

**Electronic supplementary material:**

The online version of this article (10.1186/s13643-018-0801-y) contains supplementary material, which is available to authorized users.

## Background

Once described as a disease of the affluent, type 2 diabetes mellitus (T2DM) has emerged as a significant non-communicable disease (NCD) that is threatening the economic, social and cultural fabric of African populations [1]. High diabetes prevalence is driven by economic expansion, urbanisation, lifestyle choices and ageing [2, 3], with significant variation from one geographical region to the other [4]. The regions include countries of the same or different lingua franca (e.g. Francophone vs. Anglophone countries), as well as urban and rural areas [5, 6]. Despite the paucity of data, Africa is set to bear the most significant T2DM epidemiological burden including its complications and mortality in the coming decades [7, 8]. By 2045, Sub-Saharan Africa (SSA) alone is projected to experience an approximately 156% increase in diabetes prevalence compared to 48% of the world [3].

According to the global burden of disease study, NCDs including diabetes contribute yearly to more than 60% of the global mortality, 80% of death, as well as 54% of global disability-adjusted life years (DALY) lost [1, 9, 10]. Of these, 90% of preventable death and 80% of early death occur in developing countries including the African region [1, 10]. In Africa, the burden of diabetes and other NCDs such as heart diseases are expected to eclipse that of communicable disease by 2025 in terms of associated mortality and morbidity [6, 11, 12]. Yet, communicable diseases attract the most political attention and resource allocation [13–15]. While this double burden of disease is evident, Africa does not have the resources and infrastructure to deal with the growing burden of diabetes, in addition to existing communicable diseases [15]. This double burden of disease culminates in a cascade of challenges faced by policy makers, health professionals and funders alike, with significant preventative medicine and public health dimensions. Additionally, 69.2% of the population aged 20 to 79 years not only are undiagnosed and unaware of its complications in SSA alone [3] but also unable to access the correct educations and health services [16]. Consequently, the majority of patients are diagnosed after presenting complications.

While knowledge of diabetes risk factors is important to positively shift population distributions, no systematic review and meta-analysis, to our knowledge, has examined the strength of associations between these risk factors and T2DM in Africa as well as the role of urban and rural areas. Identifying the most important diabetes risk factors may (1) influence policies and improve the allocation of resources and (2) substantially halt diabetes incidence, increase health gains and prevent loss of productivity due to complications and premature death [1, 17]. However, since we cannot affect T2DM derived from non-modifiable risk factors such as family history, sex and age [18, 19], the study considers modifiable risk factors within the African context and aims to conduct a systematic review and meta-analyses of all available published data and qualified studies that have examined these risk factors and T2DM in Africa.

## Review of diabetes modifiable risk factors: the African context

Despite a significant paucity of empirical data in Africa, some advances in diabetes risk factor studies have been made. Yet, apart from genetic factors and predispositions of diabetes in Africa [20], diabetes natural history, as well as its clinical significance are poorly understood [20, 21]. This is likely due to the fact that the majority of the risk factor studies have been conducted within Western countries [21, 22]. As such, researchers still remain somewhat unsure as to what extent much of the existing knowledge applies to Africa [21]. For modifiable risk factors, the broader determinants such as social, cultural, environmental and economic influences are likely to be Africa-specific [21]. Here, we discuss these specific risk factors with respect to T2DM, and they include diet, tobacco, alcohol, physical inactivity, and adiposity [6, 14].

## Diet

Around the globe, the epidemics of adiposity (e.g. overweight/obesity) and T2DM are mainly driven by the quality of diet and disproportionate caloric consumption [23]. Evidence suggests that independent of BMI, fat quality and carbohydrates are important predictors of diabetes [24]. For example, while glycaemic load and trans fat are strongly associated with increased risk of diabetes [23], higher consumption of high-fibre diet including fruits and vegetables are associated with decreased diabetes risk. This is exemplified in a meta-analysis that suggests that two servings of whole grain per day is associated with a 21% lower risk of diabetes [25]. In Africa, studies on dietary patterns conducted in urban Ghana by Frank et al. [26] showed inadequate fruit and vegetable consumptions association with increased risk of T2DM. These findings are concordant with Ekpenyong et al.’s [27] study among Nigerian population and also reported in Sack et al.’s [7] study among a Senegalese population. In North Africa countries such as Egypt, key diets are mainly rich in high glycaemic load and high glycaemic index, including white bread and polished rice [28]. The high intake of trans fat is now evident in Africa countries. Egypt is now among the world highest consumers of these unhealthy fats [28].

In Africa, due to food market globalisation, multinational fast food chains have multiplied substantially, contributing to the transition from a high-fibre diet to an energy-dense westernised diet [29, 30]. For example, within SSA alone, fast food outlets have increased from zero in 1980 to the current figure of more than 1000 Kentucky Fried Chicken outlets [1, 31]. There are now about 900,000 retail Coca-Cola outlets across SSA alone, with approximately 78 million servings consumed daily [1, 32]. Independent of BMI, consumption of sugar-sweetened beverages increases the risk of T2DM [23]. The most recent meta-analysis showed that subjects who consume one to two servings of sugar-sweetened beverages per day fall in the higher quartile and have 26% greater risk of diabetes than those in the lower quartile [23, 29]. Further, studies suggest that a 1% rise in sugar-sweetened beverages contributes to an additional 4.8% overweight, 2.3% obese and 0.3% diabetic adults [33].

In many Africa countries, fruit and vegetable availability are seasonal. As such, most African’s fruit and vegetable consumptions are not incorporated into normal daily food routines [34]. While the food market globalisation has the propensity to boost the availability of fruit and vegetables, easy access is strongly associated with higher socioeconomic positions in most countries and ultimately determines their intake [35].

## Tobacco

Tobacco use (either smoked or smokeless) has a strong relationship with T2DM, either as an independent risk factor or in clusters with other risk factors such as centripetal obesity [36, 37]. Smokeless tobacco products are the leaves of a plant called *Nicotiana tabacum* [38, 39], which are considerably cheaper than cigarettes [40] and used more by people of low socioeconomic status. The product is consumed as a smoke, a chew or a snuff. A meta-analysis by Willi et al. [41] shows that smokers have a 45% increased risk of diabetes compared with non-smokers. Smoking is very common among men in both SSA and North Africa including Tunisia and Libya. In Egypt, approximately, 39.7% of adult males are smokers [28]. In Africa, despite a paucity of data on smokeless tobacco toxicities, studies that do exist show that traditionally made smokeless tobacco has higher carcinogenic tobacco-specific nitrosamines than commercially made cigarettes smoking [39, 42]. In South African studies, women who are heavy smokers showed increased diabetes risk [39].

Prevalence of smokeless tobacco use among children aged between 13 and 15 years, in some African countries between 2007 and 2009, was 4.6% (boys 4.6%; girls 4.3%) in Tanzania (Dares Salaam), 22.2% (boys 18.9%; girls 24.5%) in rural Sierra Leone, 20.8% in Democratic Republic of Congo (Kinshasa), 21.9% in Gambia (Banjul) and 5.1% (boys 5.4%; girls 4.4%) in Cameroon (Yaounde) [39, 43]. The use of smokeless tobacco is widespread, yet under-researched [44]. Due to the globalisation of the tobacco industry’s influence [40, 45] and the persistently low level of health literacy, tobacco prevalence (both smoked or smokeless) is expected to reach pandemic proportions in Africa within the next decade [40, 46].

## Alcohol

Alcohol use is a major contributor to premature death and disability [47]. A systematic review by Howard et al. [48] found moderate alcohol consumption is associated with a decreased incidence of T2DM. Alcohol abuse vastly increases adiposity and abdominal obesity among all sexes [48]. Studies conducted in Nigeria [49], Kenya [50], South Africa [51] and other African countries [16] found varied correlations between alcohol consumption and diabetes. Others have shown alcohol abuse to be strongly linked to diabetes incidence [52]. These findings have been reported among different population groups in Africa, including rural South Africa [53], Kenya [54] and Nigeria [52, 55].

Alcohol use is high in Africa, except in countries where it is prohibited. In many African countries, alcohol is central to the cultures, traditions, customs and social life, with long-standing historical significance [56, 57]. Western spirits, although expensive, are culturally and economically important due to their status [58]. Western spirits are chiefly reserved for traditional drinking events and major occasions [59]. However, locally produced alcohol is common and cheaper and is most preferred [58, 59]. It is widely consumed both in rural areas and poor urban cities [60] and includes fermented beverages like *ogogoro* in Nigeria, *burukutu* and *pito* or *ginlike* (mainly illicit) in Zambia and Ghana or *gongo* in Tanzania [58]. Despite the growing interest in alcohol use in Africa, little is known about the patterns and levels of consumption among these populations [59]. Exacerbating the effect of alcohol use is the evolution of globalisation, acculturation and urbanisation of alcohol consumption in Africa [6], including increased ease of access [30].

## Physical inactivity

Physical activity is the major determinant of energy expenditure—a significant factor in energy balance and weight control [61]. According to WHO 2010, moderate physical activity (approximately 150 min per week) reduces the risk of diabetes by 27%, colon cancer by 21–25% and ischemic heart disease by 30% [61]. In Africa, studies presenting findings on physical inactivity are well documented. They include Nyenwe et al.’s [52] study among Port Harcourt, Nigeria populations in which physical inactivity was significantly associated with higher prevalence of T2DM. This finding is also reported by Sack et al. [7], Motala et al. [53], Assay et al. [62] and Christensen et al. [54]. Various studies conducted in SSA including Ouagadougou (Burkina Faso) [63], Cameroon [64], South Africa [65] and West Africa [66] have all linked physical inactivity to the rising burden of diabetes.

From a cultural perspective, structured or deliberate physical activity in most African countries is not usually viewed as a health-related risk factor but mainly through the lens of sports [67]. Additionally, in most countries particularly in North Africa, such as Egypt, exercise is avoided in public places, representing a significant factor in reduced physical activities [28]. There is also a scarcity of exercise facilities in Africa, and those that exist are very expensive [28]. In addition, the link between vitamin D deficiency and increased risk of diabetes is evident [68]. However, exposure to vitamin D via sunlight is reduced by traditional female clothing mainly among women of Islamic faith in many areas of Africa.

Furthermore, while physical activity is obtained from its occupational (e.g. manual labour and farming) or incidental use (e.g. walking) in most countries, the growing use of mechanisation is evident in growing urbanisation [16]. While driving in industrialised countries has largely displaced physical activity (e.g. walking and manual labour), this phenomenon is also now evident in most African countries [16, 23], particularly in cities. In cities, the growing use of technologies such as mobile phones and prolonged viewing of television further contributes to the decline in physical activity. As a result, the incidental and occupational physical activities which were once used to offset the glycaemic load and trans-fat are diminishing [23].

## Adiposity (obesity)

Historically, being overweight was once rare in Africa, due to food scarcity and high-energy expenditure [12]. However, as a result of economic, social, cultural, psychological and biological influences, obesity has soared [69]. Higher prevalence is marked by urbanisation and economic expansion [6]. For example, countries with advanced economies [70], including South Africa and Seychelles, have higher obesity prevalence [1, 6]. Gender differences in obesity prevalence are also evident. Studies conducted in other parts of Africa such as rural parts of Cameroon showed that, over a 10-year period, obesity increased by 84% and 54% among men and women respectively [71]. Obesity among men ranges from 12% and 13.8% in Ethiopia [72] and Cameroon [71] and among women 10.8% to 34% in Ethiopia [73] and Ghana [74], respectively. In addition, from a socio-cultural perspective, obesity is revered. This notion is rooted in the fundamental beliefs and traditional orientations that influence people’s perceptions and attitudes towards large body size. As a consequence, in much of Africa, being overweight is not considered as a risk factor but rather a sign of high socio-economic standing and beauty [75].

In Africa, numerous studies presenting findings on various parameters of adiposity such as obesity, overweight, abdominal obesity, BMI and waist-to-hip ratio are marked. For example, in Motala et al.’s [53] study of 1025 subjects (815 women) among the rural South African population, the multivariate analysis shows that the significant independent risk factors associated with diabetes include waist circumference (odds ratio 1.1) and hip circumference (0.9) for both men and women. These findings are in direct agreement with other studies in the African region [76–80]. Similarly, in Isara and Okundia’s [81] study among adult residents of rural communities in southern Nigeria, overweight/obesity (OR = 3.53) was significantly associated with diabetes. Kari et al. [82] also found that diabetes was associated with overweight/obesity (OR = 3.02/4.43). Among a Senegalese population, Sack et al. [7] found that abdominal obesity (OR = 1.17, *p* = 0.05) was strongly associated with diabetes. Similar findings have been reported in populations of South Nigeria [27], South Africa [83] and Tanzania [22]. The association between BMI and diabetes in different parts of Africa was also evident in studies conducted in Nigeria [52], South Nigeria [27], South Africa [53] and Sudan [77].

## Psychosocial factors

Several studies have identified psychosocial factors (e.g. stress, depression, anxiety) as emerging risk factors for diabetes and NCDs [6, 14, 23, 84]. Stress is a major contributing factor in metabolic syndrome [85]. Stress not only increases the risk of developing T2DM but also increases the risk of other cardiovascular diseases [86, 87]. A systematic review and meta-analysis by Chida and Hamer [88] found a detrimental association between adverse psychosocial factors and the prognosis of diabetes. A study among South Africa women, on psychosocial factors, found that stress was strongly associated with diabetes [88]. Multiple studies conducted in Africa have associated stress to cardiovascular diseases [14]. In Africa, little data exist on rates of depression and its correlates; however, its effects must not be discounted in efforts to curb diabetes incidents.

In sum, while waiting for more empirical data and studies on diabetes risk factors to become available, existing and accessible studies may be an effective resource in understanding the contribution of these risk factors and burden of diabetes in Africa.

## Rational

In Africa, despite the primary epidemiological studies presented above, to date, no studies to our knowledge have systematically meta-analysed these findings. It is imperative to note, though, that ample reviews [12, 18, 89, 90] exist, including a limited number of meta-analysis [91, 92] predominantly focused on the epidemiological burden of diabetes (e.g. prevalence, incidence, complications, DALY and economics) and their outcomes (mortality and morbidity) [1, 8, 15, 16, 18, 20, 22, 28, 67, 89, 92–94]. The present systematic review and meta-analysis will endeavour to fill this gap, generate new hypothesis and attempt to promote a renewed attention to the role of contemporary diabetes modifiable risk factors in Africa.

## Research aim

The aim of this systematic review and meta-analysis is to assess the association between modifiable risk factors and T2DM in Africa.

## Objectives

1. To assess the magnitudes of associations between modifiable risk factors (obesity, physical activity, tobacco, smoking, fruit and vegetable consumption, psychosocial factors (e.g. stress, anxiety) and T2DM in African countries)
2. To use ranking to assess which risk factors have the strongest association with T2DM in Africa
3. To examine the impact of the following moderators on associations between modifiable risk factors and diabetes: study setting (rural/urban) and study country language (Anglophone/Francophone)

## Hypotheses

Guided by the findings of previous meta-analysis and systematic reviews, the hypotheses of this study include:

1. Studies on modifiable risk (obesity, BMI, physical inactivity, fruit and vegetable consumption) and their strength of associations with T2DM as an outcome will be limited.
2. Studies on tobacco consumption including smokeless tobacco and its association with T2DM will be the most limited.
3. Diabetes risk factors such as obesity and physical inactivity will be the major risk factors. Obesity will be most strongly associated with T2DM compared with other modifiable risk factors.
4. Moderators such as urban settings will have a significant influence on diabetes risk factors and T2DM.
5. Relationships between risk factors and T2DM may differ between Africa and developed countries.
6. The most important risk factor causing most burdens may differ between Africa and developed countries.

## Methods and design

### Design

The study will comprise a systematic review and meta-analysis. The study will follow the reporting guidelines and criteria set in Preferred Reporting Items for Systematic Reviews and Meta-Analysis Protocols (PRISMA-P) (Table 1).

**Table 1:** Inclusion and exclusion criteria

Inclusion criteria	Exclusion criteria
• Only empirical research from published and grey literature that have examined modifiable risk factors with T2DM as an outcome measure will be included in this study. • All studies published from 2000 to 16 November 2017 will be included to coincide with the Millennium Development Goals (MDG) launch. Also, since the WHO diagnostic criteria for diabetes was widely accepted from 1998 [18, 90] and revised in 1999, studies conducted earlier may have used different criteria for diabetes diagnosis [95]. • Only studies reporting quantitative data will be included. These may include cross-sectional, cohort, longitudinal and case-control studies. • Studies will be included, if fasting blood plasma (FPG) and oral glucose tolerance test (OGTT) were measured in accordance with the WHO criteria [96]. • The most recent study from a duplicate publication of the same data will be considered. • Only studies published in English will be included.	• Studies with impaired glucose tolerance (IGT) and impaired fasting glucose (IFG) as outcome measures will be excluded from this study. • Risk factor definitions that do not correspond to the WHO criteria will be excluded. • Reviews, commentaries and letters will be excluded. • Studies that are rated as low quality in the risk of bias assessment will be excluded.

### Heterogeneity in measured outcomes

The literature from this area suggests that an amount of heterogeneity may exist, such as differences in methodological approaches making study comparison difficult, and some may lack clear case definitions, standard diagnostic criteria or control groups to evaluate potential study biases [6]. Most physical inactivity studies may use self-report questionnaires that are not validated [64, 95], while others may use locally validated self-reported questionnaires [67, 83, 96]. As a result, variations of the effect in both primary and secondary outcomes are possible.

### Primary outcomes

The study will provide a summary of pooled measure of association between one or more risk factors and T2DM and rank them accordingly.

### Secondary outcome

The study will provide more reliable evidence in the understanding of the contemporary diabetes modifiable risk factor associations with T2DM in Africa.

### Database searches

#### Health and biomedical

- CINAHL, MEDLINE, PsycINFO, Psychology and Behavioural Science, Global Health and Embase

#### Grey literature

- Dissertation of Thesis
- Conferences
- BIOSIS Previews, Embase, MEDLINE, CINAHL Plus, Grey Literature reports, PsycEXTRA, OpenGrey, WorldWideScience, World Health Organization
- International Diabetes Federation, African Journal Online, African Development Bank, UNICEF and USAID database

#### Multidisciplinary

- Scopus and Academic Onefile

#### Internet search engines

- Google, Google Scholar, research centres in Africa and university websites

Syntax of search terms and subject headings terminologies will be adapted according to the requirements of the individual databases (Additional file 1). According to chosen databases, all saved search strategies may be routinely run for any new studies that might be added.

### Statistical methods

#### Selection of studies

**Fig. 1:**
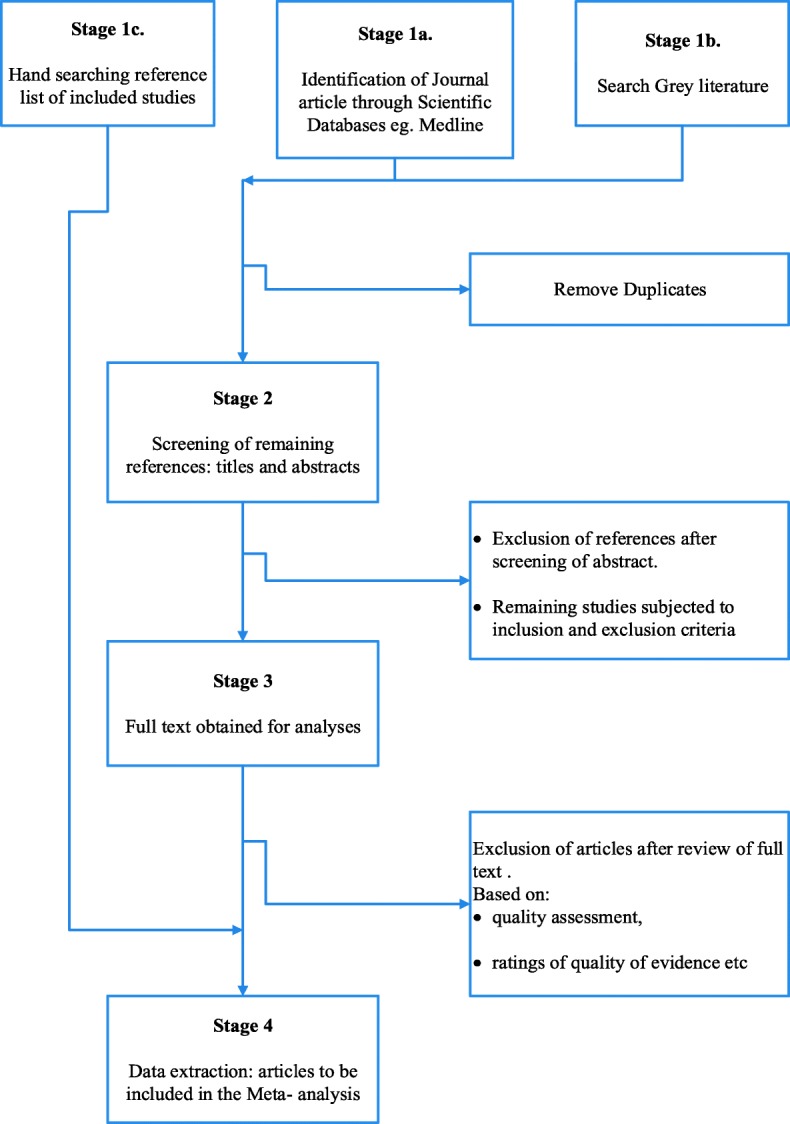
PRISMA flowchart for systematic review

Final search results will be imported to Endnote X8 and duplicates will be deleted. For multiple studies using a single dataset, the most recent will be used. Titles and abstracts will undergo a first stage of screening by two reviewers. Full text of potentially eligible studies will be obtained and screened for final inclusion (please see Fig. 1). Two independent reviewers will screen for eligibility and inclusion. A third independent author will be consulted to reach consensus, should any disagreement arise. At this third stage, reasons for exclusion studies will be well recorded.

### Data extraction, coding and management

Two reviewers will extract data directly from included studies into a Microsoft Excel spreadsheet. Any inconsistencies will be resolved by consensus and, if necessary, a third reviewer will be consulted. Extracted data will include authors, year of publication, type of publication, response rates for both published and unpublished studies, study years and study design and risk factors (obesity, BMI, physical inactivity, fruit and vegetable consumption, urban, rural, age and psychosocial factors) with T2DM as an outcome measure. Others include study location (country/nation) and sample size, odds ratio (OR), confidence interval (CI), mean and standard deviation (SD).

### Study quality and critical appraisal

#### Quality assessment

PRISMA, Critical Appraisal Skills Programme (CASP) [97] and Newcastle-Ottawa Scale (NOS) [98] will be used to determine for study quality using tools tailored to each study design. Figure 1 depicts the PRISMA flow chart.

### Data analysis

All included studies will be summarised descriptively before carrying out statistical analyses with Comprehensive Meta-Analysis (CMA) version 2 software. The analysis will synthesise the evidence to provide a summary of pooled measure of association between one or more risk factors and T2DM. The available measure of association will determine the method of collapsing and the metric that best fits. Thus, if correlation coefficients are used, other statistical measures will be converted to correlation coefficients and unadjusted odds ratios converted to correlation coefficients. Statistical measures of association between risk factors and T2DM may include regression coefficients with SD, OR, and dichotomous measures such as the χ^2^ test. Additionally, CMA software will be used to convert standardised means. We will also report two-tailed 95% CI, *p* values and ORs or SDs, then use Cohen’s ‘*d*’ to calculate the effect sizes for each risk factor. Both bivariate and multivariate associations between risk factors and T2DM will be retained; however, only bivariate associations will be used in the meta-analysis, given the challenges to meta-analysing adjusted data [99]. Associations from multivariate analyses will be discussed in-text and possibly synthesised using vote-counting methods (see [100]).

Longitudinal data will be included if data are reported for multiple time points. If all included studies focus on T2DM as an outcome, heterogeneity of the effect sizes will then be assessed using the *Q* and *I*^2^ statistics [101]. To account for the variations in the sample sizes, the CMA will be employed to calculate the weighted effect sizes [102], i.e. ensuring that more weights are given to the effects from larger samples. Considering the purpose of the review is to generalise our findings, mixed effects model will be used.

The mixed effect models will be used for moderator analysis [103] as a more conservative approach to enable the testing of differences between different moderator levels (e.g. [102]). This model will ensure that differences in study characteristics such as study designs, risk of bias/study quality, and length of follow-up are well tested. In the case where included studies report effect size disaggregation, moderation analysis will be performed on methodological variables based on the study characteristics such as location (urban and rural) and regions (Anglophone and Francophone). The moderation analysis will also be used to compare associations from multiple time (longitudinal data) and single time points (cross-sectional data) as well as for different risk factors and rank them accordingly.

### Bias/quality assessment

Publication bias will be assessed through the steps outlined below.

For studies that focus on similar outcomes, heterogeneity of effect size among studies will be assessed using *Q* and *I*^2^ statistics [101, 104]. Due to the anticipated heterogeneity described under the section titled ‘Heterogeneity in measured outcomes’, we can tentatively assign a moderate *I*^2^ statistics of above 50% cut-off heterogeneity.

1. If more than ten studies report the same risk factors, the symmetry of outcomes funnel plots will be assessed.
2. Egger’s weighted regression method will be used [98].
3. A failsafe number will be calculated to estimate the number of unlocated studies with an average zero effect size required to significantly change the results.

### Dealing with missing data

If we find that studies have not reported study designs, methods, risk factors or outcomes, corresponding authors will be contacted via email. If no further detail can be obtained, it will be narrated descriptively and identified as a study limitation.

## Discussion

This study aims to add to the extant literature by synthesising the evidence on the association between modifiable risk factors and T2DM in Africa and to provide a reliable evidence base for policy makers and future research.

### Strength and limitations of the review

This is the first known systematic review and meta-analysis of associations between TD2M and modifiable risk factors in Africa. The strengths of this study include stringent and strong adherence to the process of conducting systematic review and meta-analyses and quantitative research rigour, for example, stringent adherence to the PRISMA checklist, including a transparent approach to searching, screening, reviewing, selecting studies and data extraction [105]. The study will ensure that the search area and inclusion criteria are sufficiently complete to encompass a wide range of diabetes modifiable risk factors exposure measures and T2DM. Although we will attempt to locate as many unpublished studies as possible, the findings may be susceptible to selective reporting. Furthermore, the fact that only studies published in English language will be included may constitute a limitation.

### Dissemination plans

This systematic review and meta-analysis will form a part of the primary author’s PhD thesis. The findings will be disseminated through publications in peer-reviewed journals, conferences and presentations.

### Study registration

This systematic review and meta-analysis has been registered with PROSPERO—the International Prospective Register of Systematic Reviews, registration number: CRD42016043027.

## Additional file

Additional file 1: Search terms and strategy. (DOCX 19 kb)

## Abbreviations

BMI: Body mass index
CASP: Critical Appraisal Skills Programme
CI: Confidence interval
CMA: Comprehensive Meta-Analysis
DALY: Disability-adjusted life years
IDF: International Diabetes Federation
NCD: Non-communicable disease
NOS: Newcastle-Ottawa Scale
OR: Odds ratio
PRISMA-P: Preferred Reporting Items for Systematic Reviews and Meta-Analysis Protocols
PROSPERO: International Prospective Register of Systematic Reviews
SD: Standard deviation
SSA: Sub-Saharan Africa
T2DM: Type 2 diabetes mellitus
WHO: World Health Organization
