# Supplementary material for: Modifiable and emerging risk factors for type 2 diabetes in Africa: a systematic review and meta-analysis protocol
Source: Syst Rev. 2018 Sep 12;7:139. doi: 10.1186/s13643-018-0801-y (PMC6136189; doi:10.1186/s13643-018-0801-y)
Supplement: Supplementary file 1 — Search terms and strategy. (DOCX 19 kb) [file 13643_2018_801_MOESM1_ESM.docx]

MeSH terms combined by Boolean Commands ‘AND’ and ‘OR’

**Search terms and strategy**

Diabet* OR T2DM*

AND

“Risk factor*” OR “Physical activ*” OR “Physical inactivit*” OR Smok* Or Nicotin* OR Snuff* OR diet* OR psychosocial* OR depress* OR Stress* OR Anxiet* OR Tobacco* OR Cigarett* OR alcohol* OR Fruit* OR Vegetable* OR “Body Mass Index*” OR BMI* OR Overweight* OR Obes* OR Urban* OR rural*

**AND**

Algeria* OR Angola* OR Benin* OR Botswana* OR “Burkina Faso*” OR Burundi* OR “Cape Verde*” OR Cameroon* OR Chad* OR Comoros* OR Congo* OR Djibouti* OR Egypt* OR “Equatorial Guinea*” OR Eritrea* OR Ethiopia* OR Gabon* OR Gambia* OR Ghana* OR “Guinea-Bissau*” OR “Ivory Coast*” OR Kenya* OR Lesotho* OR Liberia* OR Libya* OR Madagascar* OR Malawi* OR Mali* OR Mauritania* OR Mauritius* OR Morocco* OR Mozambique* OR Namibia* OR Niger* OR Rwanda* OR “Sao Tome and Principe*” OR Senegal* OR Seychelles* OR “Sierra Leone*” OR Somalia* OR “South* Africa*” OR Sudan* OR Swaziland* OR Tanzania* OR Togo* OR Tunisia* OR Uganda* OR Zambia* OR Zimbabwe* OR “Central Africa*” OR “West* Africa*” OR “East* Africa*” OR “North* Africa*” OR “sub Saharan Africa*” OR Afric*
